# Trypanocidal activity of the proteasome inhibitor and anti-cancer drug bortezomib

**DOI:** 10.1186/1756-3305-2-29

**Published:** 2009-07-07

**Authors:** Dietmar Steverding, Xia Wang

**Affiliations:** 1 BioMedical Research Centre, School of Medicine, Health Policy and Practice, University of East Anglia, Norwich, NR4 7TJ, UK

## Abstract

The proteasome inhibitor and anti-cancer drug bortezomib was tested for *in vitro *activity against bloodstream forms of *Trypanosoma brucei*. The concentrations of bortezomib required to reduce the growth rate by 50% and to kill all trypanosomes were 3.3 nM and 10 nM, respectively. In addition, bortezomib was 10 times more toxic to trypanosomes than to human HL-60 cells. Moreover, exposure of trypanosomes to 10 nM bortezomib for 16 h was enough to kill 90% of the parasites following incubation in fresh medium. However, proteasomal peptidase activities of trypanosomes exposed to bortezomib were only inhibited by 10% and 30% indicating that the proteasome is not the main target of the drug. The results suggest that bortezomib may be useful as drug for the treatment of human African trypanosomiasis.

## Findings

Human African trypanosomiasis or sleeping sickness is a fatal disease caused by the protozoan parasite *Trypanosoma brucei*. The parasites live and multiply extracellularly in the blood and tissue fluids in the human host and are transmitted by the bite of infected tsetse flies (*Glossina *spp.). Millions of people living in 36 sub-Saharan countries are threatened with the disease and the estimated number of infected people is currently between 50,000 and 70,000 [1]. There are only four drugs available for chemotherapy of sleeping sickness and all show some degree of toxic side effects [2]. In addition, drug resistance in *T. brucei *is an increasing problem [3,4]. Moreover, at the turn of the millennium, the production of anti-sleeping sickness drugs was under threat as their manufacture was not profitable [5]. Thus, new strategies for the development of new drugs for treatment of sleeping sickness are urgently needed.

**Figure 1:**
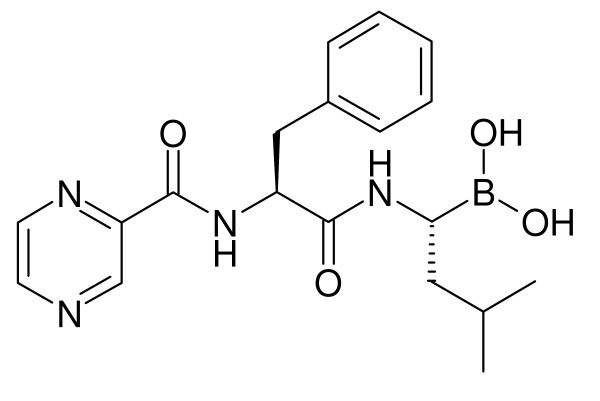
**Chemical structure of bortezomib**. Bortezomib ([(1R)-3-methyl-1-({(2S)-3-phenyl-2-[(pyrazin-2-ylcarbonyl)amino]propanoyl}amino)butyl]boronic acid) is marketed as Velcade^® ^by Millennium Pharmaceuticals and currently used to treat people with multiple myeloma.

One route for the discovery of new anti-sleeping sickness drugs is the screening of existing drugs for trypanocidal activities [6]. For example, agents that have been developed as potential anti-cancer drugs could also be of use against human African trypanosomiasis as has been shown for the ornithine decarboxylase inhibitor eflornithine [7]. Proteasome inhibitors represent a new class of anti-cancer drugs and have recently been shown to display promising anti-trypanosomal activities [8-12]. In this study, we investigated the effect of the proteasome inhibitor bortezomib (Fig. 1) on bloodstream forms of *T. brucei*. Bortezomib (Velcade^®^) is a novel chemotherapeutic agent for multiple myeloma and mantle cell lymphoma, and was approved by the FDA for treatment of these malignancies in 2003 and 2006, respectively [13].

The trypanocidal activity of bortezomib was determined with *T. brucei *bloodstream forms 427-221a while the general cytotoxicity of the drug was evaluated with human myeloid leukaemia HL-60 cells. The tests were analysed by the Alamar Blue assay as described previously [14]. In brief, cells were seeded in 24-well plates in a final volume of 1 ml of appropriate culture medium (trypanosomes: Baltz medium plus 16.7% heat-inactivated foetal bovine serum [15]; HL-60 cells: RPMI 1640 plus 16.7% heat-inactivated foetal bovine serum) containing various concentration of bortezomib and 1% DMSO. Wells containing medium and 1% DMSO served as controls. The initial densities were 10^4^/ml for trypanosomes and 10^5^/ml for HL-60 cells. After 24 h incubation, 100 μl Alamar Blue (11.11 mg resazurin sodium salt in 100 ml PBS) was added and the cells were incubated for a further 48 h so that the total incubation time was 72 h. Then, the plates were read on a microplate reader using a test wavelength of 570 nm and a reference wavelength of 630 nm. The 50% growth inhibition (GI_50_) values, i.e. the concentration of the drug necessary to reduce the growth rate of trypanosomes and HL-60 cells by 50% to that of controls, was determined by linear interpolation according to the method described in [16]. The minimum inhibitory concentration (MIC) values, i.e. the concentration of the drug at which all trypanosomes and HL-60 cells were killed, was determined microscopically.

**Figure 2:**
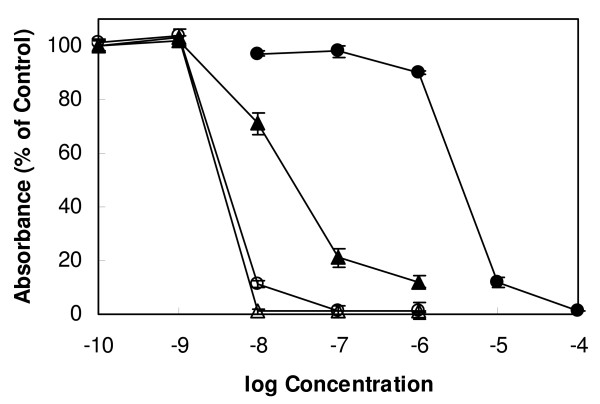
**Effect of bortezomib and melarsoprol on the growth of bloodstream forms of *T. brucei *and human myeloid leukaemia HL-60 cells**. Trypanosomes (open symbols) and HL-60 cells (closed symbols) were incubated with varying concentrations of bortezomib (triangles) and melarsoprol (circles). After 72 h of culture, cell viability and proliferation were determined with the colorimetric dye resazurin. Mean values ± SD of four experiments are shown.

Bortezomib showed a dose-dependent effect on the growth of *T. brucei *bloodstream forms with a GI_50 _value of 3.3 nM and a MIC value of 10 nM (Fig. 2). For comparison, the trypanocidal activity of the commercial anti-sleeping sickness drug melarsoprol was determined under the same experimental conditions. Melarsoprol exhibited a GI_50 _value of 3.8 nM and a MIC value of 100 nM (Fig. 2). Thus, the trypanocidal activity of bortezomib was similar to melarsoprol with respect to the GI_50 _value and was 10 times greater with respect to the MIC value. As can be expected from a drug to treat hematologic malignancies, bortezomib exhibited also cytotoxic activity against HL-60 cells with a GI_50 _value of 26.7 nM and a MIC value of 100 nM (Fig. 2). The GI_50 _value of bortezomib for HL-60 cells is within the range of the average GI_50 _value of 7 nM for the drug across 60 tumour cell lines (including HL-60 cells) [17]. Melarsoprol, on the other hand, was much less toxic to HL-60 cells, displaying a GI_50 _value of 3260 nM and a MIC value of 10,000 nM (Fig. 2). As a result, the GI_50 _and MIC ratios of cytotoxic/trypanocidal activities (selectivity indices) for bortezomib were less favourable (GI_50 _ratio = 8; MIC ratio = 10) than that of the anti-sleeping sickness drug melarsoprol (GI_50 _ratio = 860; MIC ratio = 100).

To check whether the trypanocidal activity of bortezomib is due to inhibition of the proteasome, the activity of this multi-enzyme complex in trypanosomes exposed to the drug was determined. In this experiment, 10^7 ^trypanosomes/ml were incubated with or without 100 μM bortezomib for 2 h. After washing three times with PBS/1% glucose, the trypanosomes were lysed in 10 mM Tris, 2 mM ATP, 0.1 mM EDTA, pH 7, 1 mM DTT, 0.2% NP-40 and centrifuged at 16000 × *g*. The chymotrypsin-like activity and the trypsin-like activity of the clarified supernatant were assayed in 50 mM HEPES, pH 7.5 with 10 μM Suc-LLVY-AMC and 10 μM Z-GGR-AMC, respectively. Surprisingly, bortezomib inhibited the chymotrypsin-like activity and the trypsin-like activity of the proteasome in trypanosomes only by 30% and 10%, respectively. Under the same experimental conditions, the drug inhibited the proteasomal chymotrypsin-like and trypsin-like activity in HL-60 cells by 100% and 90%, respectively. These findings indicate that the trypanocidal action of bortezomib is most likely not the result from inactivation of the proteasome.

**Figure 3:**
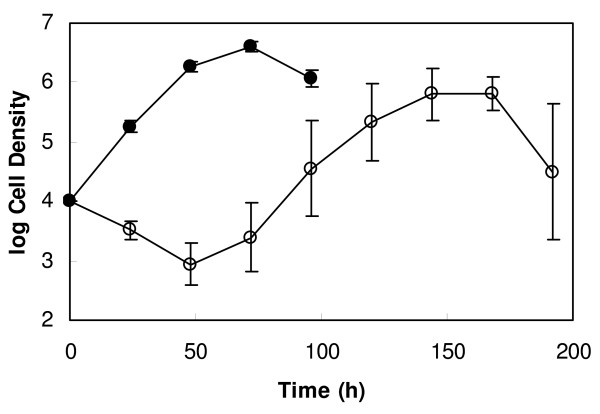
**Effect of pre-incubation with bortezomib on the growth of bloodstream forms of *T. brucei***. Trypanosomes (5 × 10^5^/ml) were incubated in the presence (open circles) or absence (closed circles) of 10 nM bortezomib for 16 h. Thereafter, trypanosomes were diluted to a cell density of 10^4^/ml in fresh medium and further incubated. At the indicated time points, trypanosomes were counted using a Neubauer haemocytometer. Geometric means ± SD of four experiments are shown. In control cultures, parasites died between 96 and 120 h of cultivation as has been shown previously [20]. In bortezomib-treated cultures, parasites died between 192 and 264 h of cultivation.

For treatment of multiple myeloma, the recommended dose and treatment schedule of bortezomib is 1.3 mg/m^2 ^administered as a 3 to 5 second bolus intravenous injection on days 1, 4, 8 and 11 of a three week cycle, for up to 8 cycles [18]. Pharmacokinetic/pharmacodynamic studies showed that on day 11 the mean plasma concentration of bortezomib falls from 422 nM to 10 nM within 16 h of administration of the drug [19]. As 10 nM is the MIC value of bortezomib, we wanted to investigate whether exposure of trypanosomes to 10 nM of the drug for 16 h is long enough to kill the parasites. To this end, 5 × 10^5 ^trypanosomes/ml were treated with or without 10 nM bortezomib for 16 h, then diluted to 10^4 ^cells/ml in fresh medium for further incubation and counted every 24 h using a Neubauer haemocytometer. For the first 48 h of re-incubation, the densities of cultures containing trypanosomes that had been exposed to bortezomib decreased continuously (Fig. 3). Thereafter, the numbers of trypanosomes started to increase but did not reach those of control cultures (Fig. 3). This result shows that treatment of trypanosomes with 10 nM bortezomib for 16 h leads in the killing of 90% of the parasites.

In conclusion, bortezomib has been shown to display substantial trypanocidal activity. The current therapy regime of bortezomib to treat cancer is probably not applicable for treatment of sleeping sickness. The results of this study indicate that a shorter treatment regime with a higher dosage may be appropriate. Importantly, bortezomib can be also administered subcutaneously [19] whereas most of the current anti-sleeping sickness drugs have to be given intraveneously [21]. Before developing bortezomib as an anti-sleeping sickness drug, animal experiments would need to be performed to establish the *in vivo *efficacy of this proteasome inhibitor. However, a selectivity index of 10 may be regarded as insufficient for proceeding to animal experiments. The Special Programme for Research and Training in Tropical Diseases at the World Health Organization (WHO/TDR) recommends a selectivity index of >100 to pursue such animal studies [22]. Compared with normal cells, however, the cytotoxicity of bortezomib determined for HL-60 cells may be overestimated. In addition, bortezomib may serve as a lead for the development of analogues with improved selectivity. Another possibility would be to use bortezomib in combination with the current drugs to treat sleeping sickness. Such drug combination regimes may lead to synergistic effects, in which lower amounts of drugs sufficient to kill the parasites would lead to a reduction in toxicity.

## Competing interests

The authors declare that they have no competing interests.

## Authors' contributions

XW and DS carried out the experiments. DS conceived the study and prepared the final draft of the manuscript. All authors read and approved the final manuscript.
